# Optimizing Metabolic Assessment: Maximal Fat Metabolism, Lactate Dynamics, and Cardiorespiratory Determinants at Different Pedaling Frequencies

**DOI:** 10.1155/jdr/2259315

**Published:** 2026-02-25

**Authors:** Ahmad Alkhatib

**Affiliations:** ^1^ School of Life and Health Sciences, Birmingham City University, Birmingham, England, UK, bcu.ac.uk

**Keywords:** cardiorespiratory, cycling, exercise intensity, fat oxidation, indirect calorimetry, metabolism, pedal rate, weight loss

## Abstract

**Aims/Objectives:**

Accurate metabolic exercise testing is essential for assessing cardiometabolic health in both athletes and clinical populations with prediabetes and diabetes. This study investigated whether and how fat, carbohydrates and lactate diagnostics are influenced by ergometry testing pedaling frequency.

**Methods:**

This randomized cross‐over repeated‐measures trial, examined human participants for cardiorespiratory oxygen uptake ($\dot{V}O_{2}$) and carbon dioxide production ($\dot{V}CO_{2}$), and blood lactate concentration (BLC), using two separate incremental load ergometry exercise tests until exhaustion, at higher versus lower cycling pedaling frequencies of 100 and 50 revolution per minute (RPM). Metabolic diagnostics of fatty acid oxidation (FAO), carbohydrates oxidation (CHO), maximal FAO (MFO) and associated MFO intensity (Fatmax) were estimated by stoichiometric equations and compared at 100 versus 50 RPM.

**Results:**

Higher $\dot{V}O_{2}$, $\dot{VC}O_{2}$, BLC and CHO and lower FAO were found for all submaximal intensities at 100 RPM than at 50 RPM (all *p* < 0.01). Fatmax power output was significantly lower (83.7 ± 20.3 vs. 99.8 ± 25.8 *W*, *p* < 0.05, effect size *d* = 0.70) at 100 than at 50 RPM. However, pedaling frequency‐dependent effects reflected nonsignificant changes in MFO (0.58 ± 0.16 vs. 0.52 ± 0.15 g.min^−1^, *p* = 0.12, *d* = 0.39), and also in the corresponding BLC at MFO (1.70 ± 0.45 vs. 1.30 ± 0.39  mmol.L^−1^, *p* = 0.06, *d* = 0.9).

**Conclusions:**

Metabolic assessments should prioritize absolute MFO and BLC dynamical changes over Fatmax intensities, when interpreting fat‐oxidation capacity, particularly under varying pedaling frequencies. By jointly characterizing blood‐based and respiratory‐based diagnostics under different exercise assessment conditions, this study helps improve the reliability of diagnosing the metabolic status in both healthy individuals and patients with metabolic disease.

## 1. Introduction

Accurate metabolic exercise assessment remains a central focus in addressing weight management and fat‐loss outcomes, particularly given the rising prevalence of obesity and Type 2 diabetes [1]. Standardized cycling ergometry testing commonly estimates fatty acid oxidation (FAO) and carbohydrate oxidation (CHO) via indirect calorimetry, measuring oxygen uptake ($\dot{V}O_{2}$), carbon dioxide production ($\dot{VC}O_{2}$), and the respiratory exchange ratio (RER). Two key diagnostic thresholds frequently used for prescribing exercise and evaluating metabolic health are (a) maximal fat oxidation (MFO; g·min^-1^) and (b) the exercise intensity corresponding to MFO (Fatmax), expressed relative to maximal oxygen uptake (%$\dot{V}O_{2peak}$) or power output (%P_peak_) [2–4].

Standard cardiorespiratory outcomes of $\dot{V}O_{2}$, $\dot{VC}O_{2}$, and RER of cycling ergometry exercise testing have all been previously shown to be affected by the variation of pedal frequencies at 40, 60, 80, 100, and 120 revolution per minute (RPM) [5, 6]. Higher pedal frequency resulted in higher metabolic rate at given power output, and such effects were largely explained by increases in muscle recruitment, neuromuscular function, and substrate content and synthesis [7–9]. However, despite the known pedal frequency effects on cardiorespiratory variables, and postulated effects on metabolism, it remains unclear whether and how pedal frequency impacts the important diagnostic variables of MFO and Fatmax.

Enhancing Fatmax, that is, shifting it to a higher exercise intensity while maintaining the same MFO, has shown benefits for weight loss, insulin sensitivity, and endurance performance [10–12]. For instance, 10 weeks of Fatmax‐based training improved body composition and physical fitness in overweight middle‐aged women [13]. Yet, the notion of Fatmax as the “fat‐burning zone” has been challenged, as MFO can occur across a broad range of intensities (30%–75%$\dot{V}O_{2peak}$) [14–16], and is associated with a similarly wide range of blood lactate concentrations (BLC; 0.8–2.2 mmol·L^−1^) [14]. These findings suggest that monitoring MFO in conjunction with BLC may offer more meaningful insight than relying on Fatmax alone. Previous studies have also highlighted methodological limitations affecting Fatmax, including ergometer testing stage duration and respiratory gas sampling protocols [12, 14]. Yet, no studies have independently examined how pedal frequency‐specific physiological differences influence both MFO and Fatmax.

Clinical populations with cardiometabolic conditions such as diabetes are often tested on cycling ergometry using low pedaling frequency of 50 RPM [17, 18], whereas high cadence of around 100 RPM is reported as more efficient and preferred by elite cyclists [19, 20]. However, when estimating MFO and Fatmax, studies have rarely reported their pedal frequency, with limited number reporting using 60‐80 RPM [21, 22]. Faster pedaling frequency of 100 RPM was previously shown to elevate metabolic rate and CHO reliance at the same absolute workload compared with 50 RPM, though greater FAO reliance was detected at a 100 RPM when BLC accumulation was used as the guiding parameter [7]. Building on these insights, this study is aimed at investigating how pedaling frequency (100 vs. 50 RPM) influences MFO and Fatmax, and to evaluate the joint effects on BLC.

We hypothesized that pedal frequency induced changes on indirect calorimetry measures ($\dot{V}O_{2}$, $\dot{VC}O_{2}$, and RER), and subsequently on CHO and FAO would differentially affect Fatmax compared with MFO and BLC. Specifically, we expected that higher pedaling rate would reduce Fatmax independently of changes in MFO and BLC.

## 2. Methods

### 2.1. Study Design and Participants

The study followed a randomized, repeated‐measures cross‐over design. Eleven healthy males (mean ± standard deviation [SD]; age: 23.5 ± 3.8 years, height: 1.8 ± 0.1 m, body mass: 76.6 ± 14.3 kg) provided written informed consent to participate. The study was approved by the institution′s ethics committee and conformed to the latest Declaration of Helsinki and guidelines for human testing. The sample size was calculated based on the effect of pedal frequency on blood lactate concentration and respiratory data [7], targeting a large effect size with 90% power at an alpha level of *p* < 0.05, requiring a sample of 10 participants. All tests were conducted at the same time of the day and under consistent environmental conditions (temperature: 19^°^C ± 0.8^°^C, relative humidity: 55*%* ± 9.1*%*, and barometric pressure: 1022 ± 11 mmHg).

Participants were instructed to avoid strenuous exercise or alcohol and to maintain consistent physical activity and dietary habits throughout the experimental period, including the 24 h prior to each test. Participants were university students with varied fitness levels (see Table 1 for peak data), living typical student lifestyles. During each visit, they were asked (in a standardized manner by the same physiologist) to confirm no changes in diet or activity levels during the study. Participants verbally confirmed adherence to pretest instructions on physical activity and diet before each session, and all tests were conducted by the same experienced physiologist.

**Table 1:** Peak physiological and mechanical data of cycling ergometry incremental testing at 50 versus 100 RPM.

**Peak data**	**Pedaling frequency at 50 RPM (** **m** **e** **a** **n** ± **S** **D** **)**	**Pedaling frequency at 100 RPM (** **m** **e** **a** **n** ± **S** **D** **)**
P_peak_ (*W*)	295.7 ± 45.6	298.7 ± 55.8
$\dot{V}O_{2peak}$ (mL.min^−1^)	4054 ± 561	4131 ± 682
$\dot{V}CO_{2peak}$ (mL.min^−1^)	4638 ± 618	4712 ± 743
RER_peak_	1.15 ± 0.08	1.14 ± 0.06
BLC_peak_ (mmol.L^−1^)	9.2 ± 1.9	11.0 ± 2.4^∗^

Abbreviations: BLC_peak_, peak blood lactate concentration; P_peak_, peak power output; RER_peak,_ peak respiratory exchange ratio (individually calculated as $\dot{V}CO_{2peak}$/$\dot{V}O_{2peak}$); $\dot{V}CO_{2peak}$, peak carbon dioxide production; $\dot{V}O_{2peak}$, peak oxygen uptake.

^∗^Significantly higher at 100 than at 50 RPM (*p* < 0.05).

On the day of testing, participants were instructed not to consume a heavy meal within at least 2 h before the session. Testing occurred at similar times between 08: 00 and 12: 00. Participants unfamiliar with the cycling protocol attended a prior additional familiarization session.

### 2.2. Experimental and Exercise Testing Protocol

Participants completed two incremental cycling tests at 50 and 100 RPM to exhaustion in randomized cross‐over order within a 2‐week period. All tests were performed on an electromagnetically braked cycle ergometer (Lode Excalibur Sport, Groningen, the Netherlands). Saddle and handlebar positions were recorded in the first session and replicated for the second. Initial power output was set at 1 W·kg^−1^ body mass and increased by 0.5 W·kg^−1^ every 2‐min stage. Participants cycled until volitional exhaustion, defined as the inability to maintain pedaling frequency for more than 15 s despite verbal encouragement, achieving age‐predicted maximal heart rate (220 − age), or a plateau in $\dot{V}O_{2}$.

Breath‐by‐breath measurements of $\dot{V}O_{2}$ and $\dot{V}CO_{2}$ were collected using an online gas analyzer (Oxycon Pro, Jaeger, Hoechberg, Germany). Participants respired through a mouthpiece attached to a low‐resistance, low‐dead–space turbine volume transducer (Triple *V* turbine, Hans Rudolph, Kansas, United States). Flow sensors and gas analyzers were calibrated with known gasses (16% O_2_, 5% CO_2_) and a 3‐L syringe before each test.

To measure BLC, a vasodilatory hyperemic gel (Finalgon, Thomae, Biberach, Germany) was applied to the earlobe at a consistent time for all participants. Capillary blood samples (20 *μ*L) were collected from the hyperemic earlobe within ~15 s at rest and at the end of each incremental stage. Lactate was analyzed using a calibrated analyzer (Ebio Plus, Eppendorf, Hamburg, Germany) with enzymatic amperometric techniques and 2 and 10 mmol·L^−1^ control standards.

### 2.3. Data Processing and Analysis

MFO was identified as the highest absolute FAO (g·min^−1^). Fatmax was the intensity corresponding to MFO, expressed relative to both %$\dot{V}O_{2peak}$ and %P_peak_ [2, 3]. Peak power (P_peak_) was calculated as the final completed power plus the proportion of time spent in the last uncompleted stage multiplied by the power increment [14]. Relative exercise intensities were defined as %P_peak_ and %$\dot{V}O_{2peak}$. Peak and submaximal $\dot{V}O_{2}$ and $\dot{V}CO_{2}$ values were averaged for the final 30 s of each stage in both tests. RER was calculated as $\dot{VC}O_{2}$/$\dot{V}O_{2}$. FAO and CHO were estimated using stoichiometric indirect calorimetry [23]:

(1) FAO=1.6951.701×V.O2−×V.CO2

(2) CHO=4.5853.226×VC.O2−×V.O2

MFO was individually identified as the highest absolute FAO (g.min^−1^). Fatmax was defined as the intensity corresponding to MFO, expressed relative to both % $\dot{V}O_{2peak}\;$and %P_peak_ [2, 3].

### 2.4. Statistical Analyses

Data were analyzed using SPSS v26 and reported as mean ± SD. Effects of pedaling frequency, intensity, and power output on FAO and CHO were assessed using repeated‐measures one‐way ANOVA with Bonferroni post hoc tests when ANOVA assumptions were met. Paired‐samples *t*‐tests compared peak between pedaling frequencies. Effect sizes were calculated using Cohen′s d (small ≥ 0.2, medium ≥ 0.5, large ≥ 0.8). Pearson correlation coefficients were used to explore relationships. Statistical significance was set at *p* < 0.05.

## 3. Results

### 3.1. Peak Data

Peak data of P_peak_ and $\dot{V}O_{2peak}$ or RER_peak_ (Table 1) were not significantly affected at different pedaling frequencies of 50 versus 100 RPM (Table 1). However, BLC_peak_ was significantly higher at 100 compared with 50 RPM (Cohen′s *d* = 0.8, *p* < 0.05), (Table 1). Strong positive correlations were observed between P_peak_ and $\dot{V}O_{2peak}$ at both 50 RPM (*r* = 0.96, *p* < 0.01), and 100 RPM (*r* = 0.91, *p* < 0.01).

### 3.2. Submaximal Cardiorespiratory and Metabolic Responses at 50 vs. 100 RPM

**Figure 1:**
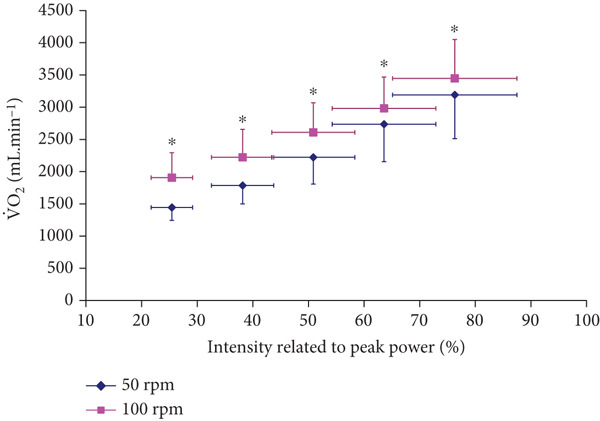
Oxygen uptake ($\dot{V}O_{2}$) responses at incremental intensities at 100 versus 50 RPM (mean ± SD).  ^∗^Significantly higher $\dot{V}O_{2}$ at 100 RPM than at 50 RPM (*p* < 0.001 for ANOVA, and *p* < 0.05 for all post hoc comparisons).

**Figure 2:**
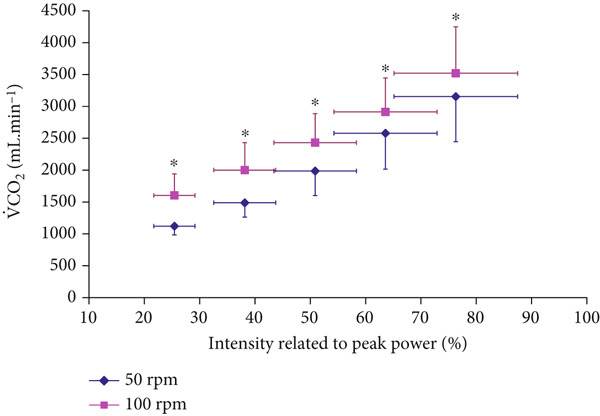
Carbon dioxide production ($\dot{V}CO_{2}$) responses at incremental exercise intensities (mean ± SD).  ^∗^Significantly higher at 100 RPM than at 50 RPM (*p* < 0.001 for ANOVA, and *p* < 0.05 for all post hoc comparisons).

Both $\dot{V}O_{2}$ and $\dot{VC}O_{2}$ levels were significantly higher at 100 RPM than at 50 RPM across all submaximal exercise intensities up to 80% of P_peak_ (*p* < 0.001, *F* = 107.011, eta squared *η* ^2^ = 0.682 for $\dot{V}O_{2}$, and *p* < 0.001, *F* = 114.832, *η* ^2^ = 0.697 for $\dot{VC}O_{2}$), which included all intensities at RER ≤ 1 (Figures 1 and 2). The correlation coefficient was significant between the conditions (*r* = 0.999 for both $\dot{V}O_{2}$ and $\dot{VC}O_{2}$ *p* < 0.0001).

**Figure 3:**
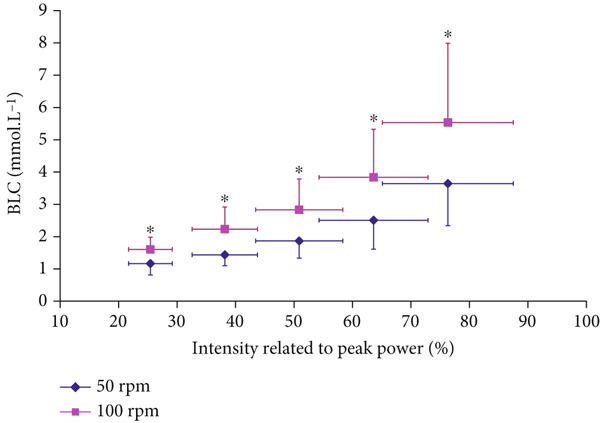
Blood lactate concentration (BLC) responses at different intensities at 100 versus 50 RPM (mean ± SD).  ^∗^Significantly higher BLC at 100 RPM than at 50 RPM (*p* < 0.001 for ANOVA, and *p* < 0.05 for all post hoc comparisons).

BLC was significantly higher at 100 compared with 50 RPM at all submaximal intensities below 80% P_peak_ (*p* < 0.001, *F* = 37.992, *η* ^2^ = 0.432) (Figure 3). The correlation coefficient was significant between the conditions (*r* = 0.990, *p* < 0.0001).

### 3.3. Fat and Carbohydrates Metabolism at 50 vs. 100 RPM

**Figure 4:**
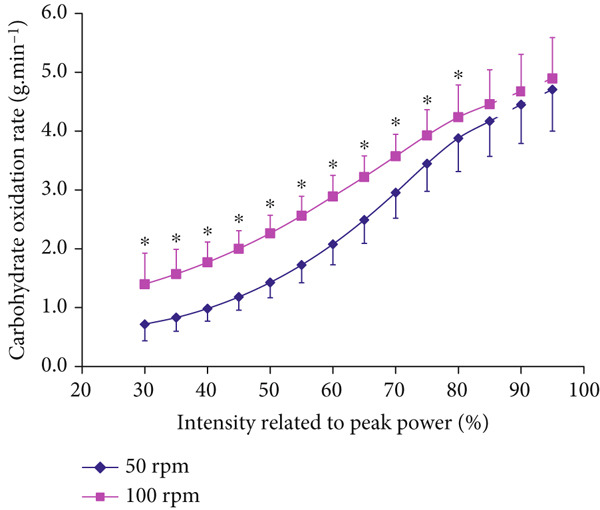
Carbohydrate oxidation responses at different intensities at 100 versus 50 RPM (mean ± SD).  ^∗^Significantly higher at 100 RPM than at 50 RPM (*p* < 0.001 for ANOVA and *p* < 0.05 for all post hoc comparisons).

**Figure 5:**
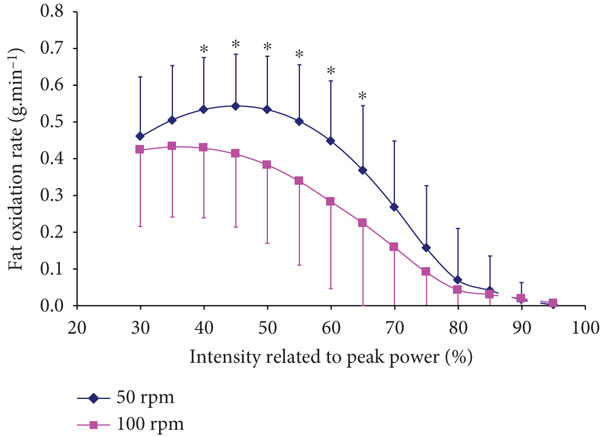
Fatty acid oxidation responses at different intensities at 100 versus 50 RPM (mean ± SD). ^∗^Significantly higher FAO at 50 than at 100 RPM (*p* < 0.001 for ANOVA, and *p* < 0.05 for all post hoc comparisons).

Higher CHO (*p* < 0.001, *F* = 21.214, *η* ^2^ = 0.683) and lower FAO (*p* < 0.001, *F* = 107.811, *η* ^2^ = 0.300) were found at 100 RPM than at 50 RPM at all submaximal intensities up until 80% and 65% P_peak_ for CHO and FAO, respectively, (Figures 4, and 5). The correlation coefficient was significant between the conditions (*r* = 0.997, *r* = 0.991, for CHO and FAO, respectively, *p* < 0.0001).

### 3.4. Pedaling Frequency Effects on Fatmax, MFO and Corresponding Power Output and BLC

MFO did not differ significantly between cycling at 50 and 100 RPM (*p* = 0.123, *d* = 0.39). BLC at MFO also showed no significant difference between the two pedaling frequencies of 50 and 100 RPM, though with a large effect size (*p* = 0.061, *d* = 0.90).

However, the power output corresponding to MFO was significantly higher at 50 RPM compared with 100 RPM (*p* = 0.045, *d* = 0.70). This was accompanied by a trend towards significantly higher Fatmax expressed as %P_peak_ at 50 RPM, which showed a large effect size (*p* = 0.056, *d* = 0.80).

When Fatmax was estimated as a function of metabolic rate of %$\dot{V}O_{2peak}$, it was significantly higher at 100 than at 50 RPM (*p* = 0.029, effect size, *d* = 0.90), (Table 2).

**Table 2:** Maximal fat oxidation and corresponding physiological responses during incremental ergometry cycling at 50 versus 100 RPM.

**Fat metabolism parameters**	**Pedaling frequency at 50 RPM (** **m** **e** **a** **n** ± **S** **D** **)**	**Pedaling frequency at 100 RPM (** **m** **e** **a** **n** ± **S** **D** **)**	**p**	**Effect size Cohen′s** **d**
MFO (g.min^−1^)	0.58 ± 0.16	0.52 ± 0.15	0.123	0.39
Power output at MFO (*W*)	99.8 ± 25.8	83.7 ± 20.3 ^∗^	0.045	0.70
BLC at MFO (mmol.L^−1^)	1.30 ± 0.39	1.70 ± 0.45	0.061	0.90
Fatmax (%P_peak_)	35.3 ± 8.1	28.4 ± 6.6	0.056	0.80
Fatmax (%$\dot{V}O_{2peak}$)	42.8 ± 7.3	48.9 ± 8.3 ^∗∗^	0.029	0.90

Abbreviations: BLC, blood lactate concentration; Fatmax (%P_peak_), maximal fat oxidation intensity relative to peak power ; Fatmax (%$\dot{V}O_{2peak}$), maximal fat oxidation intensity relative to peak oxygen uptake (%$\dot{V}O_{2peak}$); MFO, maximal fat oxidation.

^∗^Significantly lower at 100 RPM than at 50 RPM for Power output at MFO (*W*).

^∗∗^Significantly higher at 100 than at 50 RPM for Fatmax (% $\dot{V}O_{2peak}$).

## 4. Discussion

The main finding of this study is that the accuracy of metabolic assessment for the determination of maximal fat metabolism is influenced by pedaling frequency. The study found that at the same exercise intensity, cycling at 50 RPM, resulted in 20% higher Fatmax (%P_peak_), 10% greater MFO (g.min^−1^), and 16% higher power output at MFO compared with 100 RPM (Table 2). This important finding asserts that a smaller difference in absolute MFO can reflect a large difference in the corresponding exercise intensity, which is critical for metabolic assessments, especially when prescribing fat‐loss or weight management to individuals with metabolic conditions and in diabetes lifestyle interventions.

The study found that cardiorespiratory $\dot{V}O_{2}$ and $\dot{V}CO_{2}$, which are routine indirect calorimetry measurements required for estimating FAO and MFO, were both higher at 100 RPM versus 50 RPM at all submaximal intensities (Figures 1 and 2). These pedaling frequency‐effects on cardiorespiratory function were similar to higher $\dot{V}O_{2}$ and $\dot{V}CO_{2}$ at 90–100 RPM versus 40–50 RPM reported for similar submaximal intensities of 40–80 %$\dot{V}O_{2peak}$ [5, 24]. However, the study extended previous knowledge by further analyzing consequent metabolic effects on indirect calorimetry‐based relative CHO and FAO contributions, particularly at exercise intensities of 30%–65%$\dot{V}O_{2peak}$, which include the diagnostic index of Fatmax (Figures 4 and 5). Recommending “fat‐burning zone” in metabolic testing should consider pedaling frequency related effects on both respiratory data and consequent metabolic MFO and Fatmax intensities.

The study individually estimated and compared absolute amounts of MFO, which was not statistically significantly different between the two pedaling frequencies (Table 2). However, power outputs associated with MFO were 8.4% higher at 50 RPM (*p* < 0.05, *d* = 0.7) compared with those at 100 RPM. Therefore, MFO, as an absolute amount of metabolized fat, and Fatmax, as an exercise intensity should be considered independent diagnostic markers of metabolic health testing and exercise prescription. Exercise training aimed at achieving higher Fatmax resulted in improved insulin sensitivity, increased fat utilization, and fatty acids mobilizing enzymes′ activity [11, 13]. One study showed that 10‐week Fatmax intensity training has been reported to improve body composition, cardiovascular function and fitness outcomes in overweight middle‐aged women [13]. However, absolute MFO also served as a metabolic diagnostic tool irrespective of the intensity at which it occurs. For example, MFO was 18% higher in trained compared with untrained individuals, reflecting 20% higher muscle citrate synthase activity and beta‐hydroxy‐acyl‐CoA‐dehydrogenase, which are enzymes responsible for fatty acid metabolism [4]. Achieving higher MFO is influenced by several physiological determinants including muscle and fiber recruitment patterns, neuromuscular function, and glycogen content sparing and synthesis [4, 8, 9].

Potential effects on muscle and fiber recruitment patterns may partly explain pedaling frequency induced differences on MFO and BLC levels associated with MFO (~10% difference in both and with moderate effect‐size) on the one hand, and pedaling frequency‐differences induced on Fatmax intensities (~20% difference) on the other hand (Table 2). It is likely that higher pedaling frequency at given fixed exercise intensity reflected a shift towards reliance on fast‐twitch muscle fiber, combined with higher perfusion of recruited muscles with more favorable conditions for oxygen delivery [9]. This created a situation where detecting MFO at a fixed intensity relative to $\dot{V}O_{2peak}$ was nonuniform between participants. $\dot{V}O_{2peak}$ had previously been found to only explain 12% of MFO interindividual variability [16]. That study′s Fatmax of 48.3%$\dot{V}O_{2peak}$ is close to this study′s 48.9%$\dot{V}O_{2peak}$ at 100 RPM and higher than 42.8% at 50 RPM. Thus, relying on %$\dot{V}O_{2peak}\;$ alone in determining MFO changes is insufficient, whereas adding a blood‐based BLC measurement can be more sensitive in addressing the metabolic MFO interindividual variability. Joint blood‐based and respiratory‐based diagnosis helps to personalize metabolic testing and interventions patients and athletes.

Another interesting observation of the present study is that pedaling frequency alterations induced a higher Fatmax intensity related to P_peak_ (higher at 50 than 100 RPM), but such response was different when Fatmax was related to $\dot{V}O_{2peak}$ (higher at 100 than 50 RPM). This discrepancy is explained by the difference in $\dot{V}O_{2}$ required, with $\dot{V}O_{2}$ being higher at 100 RPM at any given intensity (Figure 1, Table 2). Therefore, pedaling frequency‐induced disparities in describing Fatmax at given power or given $\dot{V}O_{2}$ affect Fatmax diagnostic reliability and interpretation for both patients and athletes.

To accurately predict changes in FAO and MFO, the BLC serves as a blood‐based predictor of FAO variability (Figure 3), since it is more sensitive than intensity‐based Fatmax. Higher BLC reflects lower FAO and MFO. BLC variability was estimated to reflect a range of 0.3–0.7 g.min^−1^ of MFO variability in healthy participants, which can reach 1.7 g.min^−1^ in elite endurance athletes [14, 25]. Understanding individuals′ metabolic status through joint MFO and BLC levels is essential in the context of their joint responses to pedaling frequencies (Figures 3 and 4). Fatmax intensities alone may not be valid indicators for MFO (i.e., indicating metabolic status or weight‐loss prediction) when varying pedaling frequency. This is in line with recent debates questioning Fatmax validity when other experimental variations are present (dietary intake, nutrition supplements, and exercise intensity domains) [15, 25–27]. BLC prediction of MFO also agrees with recent emphasis on lactate role as a key signaling molecule that mediates interorgan communication through the hydroxycarboxylic acid receptor 1, which is primarily expressed in adipose tissues [28]. Further studies are needed on how BLC variability determines MFO in individual and diabetes patients.

### 4.1. Strengths and Limitations

Indirect calorimetry is the most practical method to analyze metabolic FAO, CHO, and associated MFO and Fatmax, which relies on measuring cardiorespiratory $\dot{V}O_{2}$ and $\dot{V}CO_{2}$ gas exchange, compared with other more invasive methods such as muscle biopsy and isotope tracers [3, 4]. Therefore, appropriate respiratory gas sampling protocol that provides a representation of the specific intensity domain is essential. This study averaged last 30‐s breath by breath gasses at 90–120 s of each 2‐min stage, which is consistent with achieving ≥ 95% O_2_ and CO_2_ onset kinetics steady‐state attainment, especially at low exercise intensities [29, 30]. This practical approach is more representative of the metabolic state of each increment stage than longer 3‐min stage‐protocols which averaged whole 1‐2 min samples (i.e., ≤ 95%) [31, 32]. Some may argue that longer stage increments (4‐6 min) are preferred for BLC steady state attainment due to longer duration associated with lactate transport mechanisms governing its delivery and transport compared with mechanisms associated with gas exchange [29, 32]. Studies which estimated Fatmax using longer stage durations of either 4 or 6 min, showed minimal MFO‐associated differences (~1.9 W). Thus, stage duration is unlikely to affect the pedaling frequency effects on MFO (16 W higher at 50 RPM vs. 100 RPM) (Table 2). However, longer testing durations often require multiple laboratory visits [33] that are neither practical nor tolerable by clinical populations, especially those with obesity and diabetes [10, 32]. Future studies could explore the specific disease and sex dependent differences induced by pedaling frequency.

The pedaling frequency effects on MFO and Fatmax (~10%–20% difference), and associated determinants of BLC dynamics are important in metabolic health and exercise metabolic testing. However, future studies would benefit from mathematical modeling of BLC, Fatmax, and MFO thresholds such as computing specific BLC thresholds or the whole BLC curve against CHO and FAO through the lactate‐carbohydrate model [7, 14, 34]. With a larger sample size, such modeling would particularly help in deciphering the interindividual and intraindividual variability in MFO, Fatmax and BLC at given exercise, nutrition or clinical status.

## 5. Conclusion

The joint characterization of blood‐based and respiratory‐based parameters under different pedaling frequencies within this study is essential for clinical diagnostics. This study was specifically designed to detect pedaling frequency effects on essential metabolic diagnostics of MFO, Fatmax, lactate, associated FAO, CHO, and cardiorespiratory determinants, which have not been investigated previously. Three practical messages proposed from this study (1) pedaling frequency effects on Fatmax do not necessarily reflect the changes in MFO, which may question the reliability of Fatmax as a testing metabolic diagnostic (2) pedaling frequency induced changes on FAO including MFO changes are better predicted using a blood‐based BLC levels than solely using indirect calorimetry gas‐based measurements (3) Fatmax intensity is better determined as a function of power output (%P_peak_) rather than % $\dot{V}O_{2peak}$ when varying the cycling pedaling frequency because of the postulated effects on $\dot{V}O_{2}$ and $\dot{V}CO_{2}$. Accurate metabolic diagnostics in clinical health and sports settings can be derived from this study, especially for fat‐loss and weight management.

**Nomenclature**
FAO: fatty acids oxidation
CHO: carbohydrates oxidation
$\dot{V}O_{2}$
: oxygen uptake
$\dot{VC}O_{2}$
: carbon dioxide production
RER: respiratory exchange ratio
MFO: maximal amount of fatty acid oxidation
Fatmax: intensity corresponding to maximal fat oxidation
P_peak_
: peak power output
%P_peak_
: Intensity relative to peak power output
$\dot{V}O_{2peak}$
: peak oxygen uptake
%$\dot{V}O_{2peak}$
: Intensity relative to peak oxygen uptake
BLC: blood lactate concentration
CHO: carbohydrates oxidation
RPM: revolution per minute
O_2_
: oxygen
CO_2_
: carbon dioxide
mL.min^−1^
: milliliter per minute
mL.kg.min^−1^
: milliliter per kg per minute
d: cohen′s effect size d
mmol.L^-1^
: millimolar per liter
W.kg^-1^
: Watts per kilogram
η^2^
: Eta Squared

## Ethics Statement

The study was conducted in accordance with the Declaration of Helsinki and approved by the Institutional Ethics Committee of the University of Essex (Department of Biological Sciences Ethics committee Approval: 30‐10‐2004).

## Consent

Informed consent was obtained from all subjects involved in the study.

## Conflicts of Interest

The author declares no conflicts of interest.

## Funding

No funding was received for this manuscript.

## Data Availability

The data are available on request from the author.

## References

[1] GBD 2021 Adult BMI Collaborators , Global, Regional, and National Prevalence of Adult Overweight and Obesity, 1990–2021, With Forecasts to 2050: A Forecasting Study for the Global Burden of Disease Study 2021, Lancet. (2025) 405, no. 10481, 813–838. 40049186 10.1016/S0140-6736(25)00355-1 PMC11920007

[2] Perez-Martin A. , Dumortier M. , Raynaud E. , Brun J. F. , Fedou C. , Bringer J. , and Mercier J. , Balance of Substrate Oxidation During Sub-Maximal Exercise in Lean and Obese People, Diabetes & Metabolism. (2001) 27, 4 Pt 1, 466–474, 11547220. 11547220

[3] Achten J. , Gleeson M. , and Jeukendrup A. E. , Determination of the Exercise Intensity That Elicits Maximal Fat Oxidation, Medicine and Science in Sports and Exercise. (2002) 34, no. 1, 92–97, 11782653. 11782653 10.1097/00005768-200201000-00015

[4] Nordby P. , Saltin B. , and Helge J. W. , Whole-Body Fat Oxidation Determined by Graded Exercise and Indirect Calorimetry: A Role for Muscle Oxidative Capacity?, Scandinavian Journal of Medicine & Science in Sports. (2006) 16, no. 3, 209–214, 10.1111/j.1600-0838.2005.00480.x, 2-s2.0-33646270903, 16643200. 16643200

[5] Zoladz J. A. , Rademaker A. C. , and Sargeant A. J. , Human Muscle Power Generating Capability During Cycling at Different Pedalling Rates, Experimental Physiology. (2000) 85, no. 1, 117–124, 10662901, 10.1111/j.1469-445X.2000.01840.x, 2-s2.0-0034002027. 10662901

[6] Hughes E. F. , Turner S. C. , and Brooks G. A. , Effects of Glycogen Depletion and Pedaling Speed on "Anaerobic Threshold", Journal of Applied Physiology. (1982) 52, no. 6, 1598–1607, 6809718. 6809718 10.1152/jappl.1982.52.6.1598

[7] Beneke R. and Alkhatib A. , High Cycling Cadence Reduces Carbohydrate Oxidation at Given Low Intensity Metabolic Rate, Biology of Sport. (2015) 32, no. 1, 27–33, 10.5604/20831862.1126325, 2-s2.0-84920692151, 25729147. 25729147 PMC4314601

[8] Mater A. , Clos P. , and Lepers R. , Effect of Cycling Cadence on Neuromuscular Function: A Systematic Review of Acute and Chronic Alterations, International Journal of Environmental Research and Public Health. (2021) 18, no. 15, 10.3390/ijerph18157912, 34360206. PMC8345521 34360206

[9] Skattebo Ø. , Peci D. , Clauss M. , Johansen E. I. , and Jensen J. , Increased Mass-Specific Maximal Fat Oxidation Rate With Small Versus Large Muscle Mass Exercise, Medicine and Science in Sports and Exercise. (2022) 54, no. 6, 974–983, 10.1249/MSS.0000000000002864, 35576134. 35576134

[10] Brandou F. , Dumortier M. , Garandeau P. , Mercier J. , and Brun J. F. , Effects of a Two-Month Rehabilitation Program on Substrate Utilization During Exercise in Obese Adolescents, Diabetes & Metabolism. (2003) 29, no. 1, 20–27, 12629444. 12629444 10.1016/s1262-3636(07)70003-4

[11] Venables M. C. and Jeukendrup A. E. , Endurance Training and Obesity: Effect on Substrate Metabolism and Insulin Sensitivity, Medicine and Science in Sports and Exercise. (2008) 40, no. 3, 495–502, 10.1249/MSS.0b013e31815f256f, 2-s2.0-39749099343, 18379212. 18379212

[12] Alkhatib A. , Duncan M. J. and Lyons M. , Predictors of Exercise Performance, Trends in Human Performance Research, 2010, Nova Science Publishers, 168–183.

[13] Wang J. , Tan S. , and Cao L. , Exercise Training at the Maximal Fat Oxidation Intensity Improved Health-Related Physical Fitness in Overweight Middle-Aged Women, Journal of Exercise Science and Fitness. (2015) 13, no. 2, 111–116, 10.1016/j.jesf.2015.08.003, 2-s2.0-84951096507, 29541108. 29541108 PMC5812837

[14] Alkhatib A. , Maximal Fat Metabolism Explained by Lactate-Carbohydrate Model, Physiologia. (2022) 2, 121–131, 10.3390/physiologia2040011.

[15] Kuo C. H. , Harris M. B. , Jensen J. , Alkhatib A. , and Ivy J. L. , Editorial: Possible Mechanisms to Explain Abdominal Fat Loss Effect of Exercise Training Other Than Fatty Acid Oxidation, Frontiers in Physiology. (2021) 12, 789463, 10.3389/fphys.2021.789463, 34867489. 34867489 PMC8638619

[16] Venables M. C. , Achten J. , and Jeukendrup A. E. , Determinants of Fat Oxidation During Exercise in Healthy Men and Women: A Cross-Sectional Study, Journal of Applied Physiology. (2005) 98, no. 1, 160–167, 15333616. 15333616 10.1152/japplphysiol.00662.2003

[17] Åstrand P. O. and Ryhming I. , A Nomogram for Calculation of Aerobic Capacity (Physical Fitness) From Pulse Rate During Sub-Maximal Work, Journal of Applied Physiology. (1954) 7, no. 2, 218–221, 13211501, 10.1152/jappl.1954.7.2.218. 13211501

[18] Sargent C. , Scroop G. C. , Nemeth P. M. , Burnet R. B. , and Buckley J. D. , Maximal Oxygen Uptake and Lactate Metabolism are Normal in Chronic Fatigue Syndrome, Medicine and Science in Sports and Exercise. (2002) 34, no. 1, 51–56, 11782647. 11782647 10.1097/00005768-200201000-00009

[19] Hagberg J. M. , Mullin J. P. , Giese M. D. , and Spitznagel E. , Effect of Pedaling Rate on Submaximal Exercise Responses of Competitive Cyclists, Journal of Applied Physiology. (1981) 51, no. 2, 447–451, 7263451. 7263451 10.1152/jappl.1981.51.2.447

[20] Lucia A. , San Juan A. F. , Montilla M. , Cañete S. , Santalla A. , Hernández A. , Earnest C. P. , and Pérez Ruiz M. , In Professional Road Cyclists, Low Pedaling Cadences are Less Efficient, Medicine and Science in Sports and Exercise. (2004) 36, no. 6, 1048–1054, 15179176, 10.1249/01.MSS.0000128249.10305.8A, 2-s2.0-2642518873. 15179176

[21] Opazo-Díaz E. , Corral-Pérez J. , Pérez-Bey A. , Marín-Galindo A. , Montes-de-Oca-García A. , Rebollo-Ramos M. , Velázquez-Díaz D. , Casals C. , and Ponce-González J. G. , Is Lean Mass Quantity or Quality the Determinant of Maximal Fat Oxidation Capacity? The Potential Mediating Role of Cardiorespiratory Fitness, Journal of the International Society of Sports Nutrition. (2025) 22, no. 1, 2455011, 10.1080/15502783.2025.2455011, 39881476. 39881476 PMC11784066

[22] Dunst A. K. , Hesse C. , and Ueberschär O. , Enhancing Endurance Performance Predictions: The Role of Movement Velocity in Metabolic Simulations Demonstrated by Cycling Cadence, European Journal of Applied Physiology. (2025) 125, no. 4, 895–907, 10.1007/s00421-024-05663-4, 39904799. 39904799 PMC11950142

[23] Peronnet F. and Massicotte D. , Table of Nonprotein Respiratory Quotient—An Update, Canadian Journal of Sport Sciences. (1991) 16, no. 1, 23–29, 1645211. 1645211

[24] Zoladz J. A. , Rademaker A. C. , and Sargeant A. J. , Non-linear Relationship Between O2 Uptake and Power Output at High Intensities of Exercise in Humans, Journal of Physiology. (1995) 488, pt 1, 211–217, 10.1113/jphysiol.1995.sp020959, 2-s2.0-0028973323, 8568657. 8568657 PMC1156714

[25] Noakes T. D. , Prins P. J. , Volek J. S. , D’Agostino D. P. , and Koutnik A. P. , Low Carbohydrate High Fat Ketogenic Diets on the Exercise Crossover Point and Glucose Homeostasis, Frontiers in Physiology. (2023) 14, 1150265, 10.3389/fphys.2023.1150265, 37057184. 37057184 PMC10086139

[26] Alkhatib A. , Carbohydrate and Lactate Utilisation During Exercise With and Without Yerba Maté Ingestion, Proceedings of the Nutrition Society. (2015) 74, no. OCE1, 10.1017/S0029665115000427.

[27] Romijn J. A. , Coyle E. F. , Sidossis L. S. , Gastaldelli A. , Horowitz J. F. , Endert E. , and Wolfe R. R. , Regulation of Endogenous Fat and Carbohydrate Metabolism in Relation to Exercise Intensity and Duration, American Journal of Physiology. (1993) 265, 3 pt 1, E380–E391, 10.1152/ajpendo.1993.265.3.E380, 8214047. 8214047

[28] Chavez-Guevara I. A. , Fernández-Escabias M. , Hernández-Lepe M. A. , and Amaro-Gahete F. J. , Modulation of Fatty Acid Metabolism via Lactate-HCA1 Signaling: Potential Therapeutic Implications, American Journal of Physiology. Cell Physiology. (2025) 328, no. 4, C1333–C1337, 10.1152/ajpcell.00969.2024, 40094283. 40094283

[29] Jones A. M. and Poole D. C. , Oxygen Uptake Kinetics in Sport, Exercise and Medicine: Research and Practical Application, 2005, Routledge, Taylor & Francis.

[30] Xu F. and Rhodes E. C. , Oxygen Uptake Kinetics During Exercise, Sports Medicine. (1999) 27, no. 5, 313–327, 10.2165/00007256-199927050-00003, 2-s2.0-0033039718. 10368878

[31] Achten J. and Jeukendrup A. E. , Maximal Fat Oxidation During Exercise in Trained Men, International Journal of Sports Medicine. (2003) 24, no. 8, 603–608, 10.1055/s-2003-43265, 2-s2.0-0242576385. 14598198

[32] Bordenave S. , Flavier S. , Fédou C. , Brun J. F. , and Mercier J. , Exercise Calorimetry in Sedentary Patients: Procedures Based on Short 3 Min Steps Underestimate Carbohydrate Oxidation and Overestimate Lipid Oxidation, Diabetes & Metabolism. (2007) 33, no. 5, 379–384, 10.1016/j.diabet.2007.04.003, 2-s2.0-35648993044, 17936665. 17936665

[33] Beneke R. and von Duvillard S. P. , Determination of Maximal Lactate Steady State Response in Selected Sports Events, Medicine and Science in Sports and Exercise. (1996) 28, no. 2, 241–246, 10.1097/00005768-199602000-00013, 2-s2.0-0030025305, 8775160. 8775160

[34] Lovell D. I. , Stuelcken M. , and Eagles A. , Exercise Testing for Metabolic Flexibility: Time for Protocol Standardization, Sports Medicine-Open. (2025) 11, no. 1, 10.1186/s40798-025-00825-w, 40164840. PMC11958852 40164840

